# Supplementary material for: Social validation of post-treatment outcomes of adults who stutter who participated in CARE Model treatment: replication and extension
Source: Front Rehabil Sci. 2025 May 21;6:1541059. doi: 10.3389/fresc.2025.1541059 (PMC12133745; doi:10.3389/fresc.2025.1541059)
Supplement: Supplementary file 1 [file Supplementaryfile1.docx]

**Appendix A**

*Description of Treatment Program*

Communication competence training serves as one of the four distinct components of the Blank Center CARE^™^ Model (Communication, Advocacy, Resilience, and Education). A brief summary of the 11-week (i.e., 22-session) manualized treatment protocol is provided below. Treatment consists of two 60-minute sessions per week consisting of one group session as well as one individual session. Training provided during the individual sessions provide an opportunity to review what will be covered, prepare for the activities, and debrief for the weekly group sessions, wherein participants work towards strengthening their communication skills across distinct speaking scenarios, including mock job interviews, small group interactions, impromptu icebreakers, one-on-one interactions with unfamiliar persons, and multiple presentations varied both in purpose (e.g., informative, persuasive, inspirational) and audience composition (e.g., small and large groups, familiar and unfamiliar listeners).

- **Week 1** (Sessions 1 and 2): Participants are introduced to the competencies that comprise effective communication (i.e., language use, language organization, speech rate, intonation, volume, gestures, body position, eye contact, facial affect). Participants receive focused training in effective **body positioning and gestures.** Participants complete self-ratings of these communication competencies following an impromptu small group presentation.
- **Week 2** (Sessions 3 and 4): Participants identify and describe core components of communication competence and receive focused training in effective use of **facial affect,** while simultaneously continuing to strengthen competencies addressed in the prior sessions. Participants complete self-ratings of communication competencies following a small group informative speech presentation to unfamiliar persons.
- **Week 3** (Sessions 5 and 6): Participants identify and describe core components of communication competence and receive focused training in **turn-taking and listener awareness,** while simultaneously continuing to strengthen competencies addressed in prior sessions. Participants complete self-ratings of their communication competencies following impromptu dyadic exchanges as well as impromptu small group presentations to unfamiliar persons.
- **Week 4** (Sessions 7 and 8): Participants identify and describe core components of communication competence and receive focused training in effective **vocal variety (i.e., volume, rate, intonation),** while simultaneously continuing to strengthen competencies addressed in prior sessions. Participants complete self-ratings of communication competencies following impromptu dyadic exchanges, as well as impromptu small group presentations to unfamiliar persons.
- **Week 5** (Sessions 9 and 10): Participants identify and describe core components of communication competence and receive focused training in effective **language use and organization**, while simultaneously continuing to strengthen competencies addressed in prior sessions. Participants complete self-ratings of communication competencies after completing 10+ dyadic interactions with unfamiliar persons.
- **Week 6** (Sessions 11 and 12): Participants identify and describe core components of communication competence and receive additional focused training in **stuttering openly**- that is, making no attempts to avoid stuttering, increase fluency, and/or modify moments of stuttering**.** Participants complete self-ratings of communication competencies following an open mic presentation in a public forum.
- **Week 7** (Sessions 13 and 14): Participants **review and practice all core components of communication competence simultaneously**. Participants complete self-ratings of communication competencies following a persuasive speech given to a small group and a large group of unfamiliar persons.
- **Week 8** (Sessions 15 and 16): Participants **review and practice all core components of communication competence**. Participants complete self-ratings of communication competencies after serving as an interviewee in a series of panel interviews, with potential employers across diverse professions.
- **Week 9** (Sessions 17 and 18): Participants **review and practice all core components of communication competence.** Participants complete self-ratings of communication competencies following impromptu dyadic exchanges as well as impromptu presentation to a small group of unfamiliar persons.
- **Week 10** (Sessions 19 and 20): Participants **review and practice all core components of communication competence**. Participants complete self-ratings of communication competencies following impromptu dyadic exchanges as well as impromptu presentation to a small group of unfamiliar persons.
- **Week 11** (Sessions 21 and 22): Participants **review and practice all core components of communication competence.** Participants complete a self-rating of communication competencies after providing a presentation to an audience of >200 people.
