# Supplementary material for: Social validation of post-treatment outcomes of adults who stutter who participated in CARE Model treatment: replication and extension
Source: Front Rehabil Sci. 2025 May 21;6:1541059. doi: 10.3389/fresc.2025.1541059 (PMC12133745; doi:10.3389/fresc.2025.1541059)
Supplement: Supplementary file 2 [file Supplementaryfile2.docx]

**Table 2**

*Number of Non-Overlapping Untrained Observers Serving as Raters for Each Video Sample (Condition x Timepoint x Context) Across Participants*

|  | Communication Competence | | | | Stuttering Severity | | | |  |  |
| --- | --- | --- | --- | --- | --- | --- | --- | --- | --- | --- |
| Participant | Pre-Treatment | | Post-Treatment | | Pre-Treatment | | Post-Treatment | |  | *N* |
|  | Dyad | Pres | Dyad | Pres | Dyad | Pres | Dyad | Pres |  |  |
| 1 | 17 | 12 | 12 | 12 | 15 | 12 | 12 | 14 |  | 106 |
| 2 | 16 | 12 | 15 | 15 | 13 | 15 | 13 | 13 |  | 112 |
| 3 | 15 | 15 | 13 | 13 | 15 | 13 | 11 | 13 |  | 108 |
| 4 | 10 | 13 | 16 | 15 | 14 | 14 | 13 | 15 |  | 110 |
| 5 | 13 | 12 | 14 | 12 | 13 | 13 | 15 | 14 |  | 106 |
| 6 | 14 | 16 | 14 | 15 | 12 | 16 | 15 | 14 |  | 116 |
| 7 | 14 | 16 | 15 | 16 | 12 | 11 | 15 | 14 |  | 113 |
| 8 | 15 | 16 | 12 | 14 | 14 | 12 | 14 | 15 |  | 112 |
| 9 | 15 | 16 | 13 | 14 | 15 | 14 | 15 | 15 |  | 117 |
| 10 | 12 | 16 | 15 | 14 | 12 | 13 | 14 | 14 |  | 110 |
|  |  |  |  |  |  |  |  |  |  |  |
| *N* | 141 | 144 | 139 | 140 | 135 | 133 | 137 | 141 |  | 1,110 |

*Note.* Pres = presentation.
